# Editorial: Integration of Multi-Omics Techniques in Cancer

**DOI:** 10.3389/fgene.2021.733965

**Published:** 2021-08-09

**Authors:** Geoffroy Andrieux, Sajib Chakraborty

**Affiliations:** ^1^Institute of Medical Bioinformatics and Systems Medicine, Medical Center – University of Freiburg, Faculty of Medicine, University of Freiburg, Freiburg, Germany; ^2^German Cancer Consortium (DKTK) and German Cancer Research Center (DKFZ), Freiburg, Germany; ^3^Molecular Systems Biology Laboratory, Department of Biochemistry and Molecular Biology, University of Dhaka, Dhaka, Bangladesh

**Keywords:** multi-omics, systems biology, cancer, tumor hetereogeneity, spatial transcriptome

Intra-tumor heterogeneity of cancer cells originates from discrete molecular alterations that are acquired during the spatiotemporal evolution of cancer. Molecular alterations during the cancer evolution are not restricted only to the mutational events in the genomic landscape but in addition, manifest in the form of epigenomic, transcriptional, and proteomic dysregulations. Although genomics studies have shed light into the initiation and progression of cancer, the translational impact of genomics has not been fully materialized. Although large-scale genome-sequencing studies have identified druggable driver mutations in oncogenes in up to 40% of the patients, there is an unmet challenge where only a lower fraction of cancer patients (10–15%) are benefited from genotype-matched targeted therapies using genomics-based diagnostics for a temporary period before the relapse of cancer (Massard et al., 2017; Zehir et al., 2017; Malone et al., 2020). The development of genotype-matched targeted therapies is challenging partly due to the reason that distinguishing genetic mutations that are causally liked to cancer (driver mutations) from those which are not responsible for cancer (passenger mutations) (Pon and Marra, 2015). Confounding this, many of the cancer-causing mutations are in the tumor-suppressor genes leading to the loss of function of these genes. Moreover, intra-tumor heterogeneity originating from a diverse array of clonal mutations giving rise to a mixture of multiple clones within a single tumor can contribute to tumor-plasticity which eventually leads to recurrence and resistance to therapy (Ramon et al., 2020). While the single-level Omics studies have identified various cancer-specific mutations, epigenetic alterations, and molecular subtyping of tumors based on gene/protein expression, the lack of the resolving-power of single-Omics contributes to the failure in deciphering the causal relationship between molecular signatures and the phenotypic manifestation of cancer hallmarks. In contrast, the multi-OMICS integration approaches have the ability to interrogate the cancer cells/tissues in multiple dimensions as well as have the potential to discover the underlying mechanism of phenotypic manifestations of cancer hallmarks (Chakraborty et al., 2018). Therefore to understand the intricate complexity of cancer, it is required to investigate beyond the genomics, and identify the molecular alterations in different Omics-levels by integrating multi-omics data to untangle the complexity of cancer at a systemic level.

The Research Topic entitled “Integration of Multi-Omics Techniques in Cancer” underscored the importance of integrating multi-Omics data to generate key biological insights that may lead to translational applications. The study performed by Marthong et al. reported on the methylome and transcriptome alterations in a genome-wide manner in oropharyngeal cancer patients. The authors identified both epigenetically silenced and active genes representing diverse cellular pathways encompassing interleukin signaling, Toll-like receptor signaling, osteoclast differentiation, and xenobiotic metabolism. Importantly, the study identified the epigenetic dysregulation of two transcription factors—SPI1 and RUNX1 that may contribute to the altered expression of a diverse array of target genes. Interestingly, a comparison of the TCGA head and neck cancer dataset revealed a distinct epigenetic and transcriptomic feature of oropharyngeal cancer. Further corroborating the impact of altered methylome in cancer, Wang et al. identified six DNA methylation-driven genes (MDGs) with prognostic potential in Glioblastoma (GBM) that can potentially be used to stratify patients into high- and low-risk cohorts. Interestingly, the author also showed that the identified MDGs have the power to predict the potential outcome of the immunotherapies. In conclusion, this MDG-based prognostic model may serve as an indicator to assess survival and treatment options for GBM patients. To shed light on the different mechanisms of epigenetic modifications, Zhang and Guo showed how altered epigenetic modifications tightly control the transcriptomic landscape in gastric adenocarcinoma (GC). The authors identified an additive impact of the altered epigenetic marks on the differential expression of the genes suggesting a robust epigenetic mechanism controlling the gene expression in GC patients. Interestingly, the authors revealed a significant effect of DNA methylation in absence of histone modifications indicating that the histone modifications tend to mask the effect of DNA-methylation when both modifications are present. This study provides an important insight into the interrelation of different epigenetic mechanisms and their combined impact on GC. To investigate the impact of non-coding RNAs (ncRNA), Yin et al. identified microRNA (miRNA) and long noncoding RNA (lncRNAs) markers in a pan-cancer manner. The target analysis revealed that miRNA markers have the ability to control different canonical pathways including PI3K-Akt signaling and Notch signaling pathways thereby playing an important role in cancer.

In a very intriguing study, Dicks et al. attempted to elucidate the synergy between cardiac regeneration and tumor growth. Their integrated analyses revealed that multiple genes of identical cellular pathways predominantly representing cell cycle processes are activated in both early stages of cardiac regeneration in the zebrafish model and tumor development/growth across different tumor types. This study provided evidence in support of the hypothesis that tumor cells hijack normal cellular developmental pathways to promote and maintain tumor growth (Editorial, 2007).

Although transcriptomics provides a more dynamic view of a cancer cell and however, mRNAs are now considered to be a poor predictor of protein levels. Cancer proteomics analyses are now emerging as a powerful technique to predict the therapeutic response in cancer patients particularly for immunotherapy (Chen et al., 2021). By integrating transcriptome and proteome Andrieux et al. showed that the mRNA-to-protein correlation analysis can serve as a unique approach to identify the pathways prioritized by the tumor cells at different clinical stages.

In a review, article Das et al. illustrated a plethora of cancer-specific online omics-data resources and delineated a diverse multi-omics data integration strategy to facilitate the generation of new biological insights for cancer research. In this review, the authors provide a comprehensive overview of the online multi-omics resources that are dedicated to cancer. By systematically comparing the advantages and limitations of the respective online resources, they underscored the current biological and technological challenges for multi-omics data integration and proposed possible strategies to mitigate these challenges. A number of multi-omics data integration strategies have been discussed in this review article including horizontal and vertical data integration. The former represents the integration of a single level omics-data across different studies while the latter corresponds to the integration of different levels of omics-data for the same type of samples. Horizontal data integration is currently limited by the high variability of data acquisition technologies. For instance, in case of gene expression studies combining RNA-seq with microarray data remains challenging due to the different dynamic-range and gene-coverage of these techniques demanding for the development of cross-plateform integration methods. In this context, Training Distribution Matching (TDM) has been develop to facilitae the usage of RNA-seq with microarray-based machine learing model (Thompson et al., 2016). Vertical data integration can be achieved by different strategies such as post-analysis data integration involving analysis of single omics datasets individually followed by their post-analysis multi-omics integration and integrated data analysis encompassing specialized algorithms and tools to combine pre-analyzed multi-omics data sets.

Certain unmet challenges must be overcome to materialize the promise of multi-Omics based precision onco-medicine. The limited standardization and accessibility restricted the widespread usage of Omics technologies in the clinical setting. Technical and computational advancements are required to accommodate the multi-omics techniques in routine diagnostic tests for cancer patients. Another challenge is the lack of pre-processing omics data formats limiting the usage of omics data to be integrated effectively. For instance, a diverse array of data portals provide access to cancer RNA-Seq data, such as the Gene Expression Omnibus (GEO) (Barrett et al., 2013) and the Sequence Read Archive (SRA) (Wheeler et al., 2008). One of the prominent limitations of such data portals is that these primarily serve as a repository of raw RNA-seq count data archives and do not provide the full processed RNA-seq data thereby limiting the applicability of the RNA-Seq data for non-computational biologists (Li et al., 2016). In case of proteomics data, the Proteomics Identification Database (PRIDE) (Perez-Riverol et al., 2019) provide the access to the raw LC-MS/MS spectra, but do not provide pre-processed data with protein intensity values. The pre-processing of RNA-seq and LC-MS/MS data requires advance bioinformatics skills including quality control, normalization, development of downstream analysis pipelines for analysis and visualization. Therefore development of a uniform processing algorithms applicable to the raw omics datasets will be an effective strategy to ensure accessibility of the pre-processed omics data to the biologists thereby aiding them to effectively take advantage of the vast repository of online multi-omics data.

Integration of multi-Omics data may aid the identification of personalized molecular signatures to determine the treatment regimen of individual patients in the future. However, a comprehensive understanding of the intra-tumor heterogeneity is necessary to contextualize the multi-dimensional molecular alterations during the spatiotemporal evolution of cancer. Currently, the multi-Omics integration is predominantly based on the molecular snapshot of a particular time point of the cancer state. However, understanding the temporal heterogeneity of cancer cells requires interrogating the tumor at multiple time points to elucidate the tumor trajectory and cancer cell dynamics. However, collecting samples from solid tumors at multiple time points by utilizing an invasive process (biopsy) from the spatiotemporally distinct primary and metastatic sites of a tumor is not practical. A viable solution, however, emerged for the genomics studies in the form of liquid biopsy where circulating cancer cells/DNA fragments in the blood originating from tumors are analyzed to identify the temporal molecular alterations during the evolution of the tumor (Mattox et al., 2019). Apart from temporal plasticity, cancer cells also exhibit a high degree of heterogeneity in different spatial-distinct sites. For instance, spatial transcriptomics (ST) analysis revealed that the invasive margin of Glioblastoma (GBM) harbors a distinct transcriptional signature compared to the hypoxic tumor core (Smith et al., 2020). The advent of cutting-edge techniques allowing us to interrogate tumor cells in varying space and time may broaden our understanding of the spatiotemporal tumor evolution which may ultimately lead to the discovery of stage-specific therapeutic targets to delay the progression and recurrence of cancer or even block it entirely.

## Author Contributions

All authors listed have made a substantial, direct and intellectual contribution to the work, and approved it for publication.

## Conflict of Interest

The authors declare that the research was conducted in the absence of any commercial or financial relationships that could be construed as a potential conflict of interest.

## Publisher's Note

All claims expressed in this article are solely those of the authors and do not necessarily represent those of their affiliated organizations, or those of the publisher, the editors and the reviewers. Any product that may be evaluated in this article, or claim that may be made by its manufacturer, is not guaranteed or endorsed by the publisher.
